# The ethics of community water fluoridation: Part 2 - how has the ethics of community water fluoridation been appraised in the literature? A scoping review

**DOI:** 10.1038/s41415-024-8057-5

**Published:** 2025-03-14

**Authors:** Bhavini Patel, Thomas Anthony Dyer

**Affiliations:** 41415286270001 Dental Practitioner and Alumnus, School of Clinical Dentistry, University of Sheffield, 19 Claremont Crescent, Sheffield, S10 2TA, UK; 41415286270002 Senior Clinical Teacher, School of Clinical Dentistry, University of Sheffield, 19 Claremont Crescent, Sheffield, S10 2TA, UK

## Abstract

**Aim** To examine how the ethics of community water fluoridation has been appraised in the literature.

**Method** A scoping review using an updated Arksey and O'Malley framework. Six electronic databases were searched: Medline via OVID; Scopus; Web of Science; Cochrane Library; StarPlus and Google Scholar. Grey literature and reference lists of included studies were also searched.

**Results** In total, 51 studies from 15 countries were included. Many referred to biomedical ethical principles intended for clinical rather than public health interventions. Some primarily aimed to appraise its ethics whereas others considered it as part of more general discussions on fluoridation. While most acknowledged its complexity, those more supportive of fluoridation often emphasised collective benefit and those less supportive emphasised infringement of autonomy or personal liberty and concerns about harm. Few referred to public health ethics principles and frameworks available in the literature.

**Conclusion** Different approaches have been taken to appraise the ethics of fluoridation. Frequently, these were conceived for individual medical rather than public health interventions and are inadequate to resolve tension between inevitable infringement of individual consent and collective benefit in public health. Other approaches conceived specifically for public health exist that have more utility in debates and ethical decision-making.

**Supplementary Information:**

Zusatzmaterial online: Zu diesem Beitrag sind unter 10.1038/s41415-024-8057-5 für autorisierte Leser zusätzliche Dateien abrufbar.

## Key points

- Justifying the introduction, maintenance and cessation of community water fluoridation requires the consideration of its ethics. This paper summarises the different approaches used to appraise its ethics in the literature.
- The paper reports that most studies used approaches conceived for medical rather than public health interventions. These were unable to adequately resolve the tension between the infringement of individual consent and collective benefit associated with community water fluoridation.
- The paper also identifies other multidimensional approaches conceived for public health interventions with more utility for appraising the ethics of water fluoridation.

**Supplementary Information:**

Zusatzmaterial online: Zu diesem Beitrag sind unter 10.1038/s41415-024-8057-5 für autorisierte Leser zusätzliche Dateien abrufbar.

## Introduction

In the 1930s, evidence emerged that there was an inverse correlation between naturally occurring fluoride in water and dental caries. This led to the addition of fluoride to public drinking water supplies. There is a large body of evidence that supports community water fluoridation (abbreviated to fluoridation in this paper) in terms of its effectiveness,^1^^,^^2^ cost effectiveness^3^ and sustainability,^4^ while acknowledging an increased risk of dental fluorosis.^1^^,^^2^ However, despite a lack of evidence to support them,^1^^,^^5^ there have been concerns that fluoridation may have other negative health impacts and has been linked to a range of health conditions, including some cancers. Consequently, as seen with other public health measures, fluoridation remains controversial, highly emotive, and its ethics are questioned.

Public health measures, such as fluoridation, differ from clinical interventions, as they aim to address multiple factors that determine population health, prevent disease and promote health. This involves multi-agency working and legislation and consequently, consideration of their justification requires a different approach to clinical interventions. In addition, public health's aim to improve population health and reduce inequalities, particularly for vulnerable groups within society, can generate tensions between perspectives on the rights of individuals versus the responsibility of society for its individuals or the needs of a community.^6^^,^^7^^,^^8^^,^^9^^,^^10^^,^^11^^,^^12^

The first paper in this series explored public health ethics in general but used fluoridation to illustrate the concepts and principles discussed.^13^ Public health ethics is a branch of medical ethics that focuses on population rather than individual health, while still taking the latter into account when considering whether a measure is fair and just. It aims to provide approaches that examine the justification of interventions by balancing the need to improve the health and wellbeing of populations with any perceived infringement of personal freedoms or risk of harm. Given the complexity of such decision-making, frameworks have been proposed to guide ethical deliberations which attempt to include the various relevant moral, ethical and political theories and perspectives.^10^^,^^12^^,^^14^^,^^15^^,^^16^^,^^17^^,^^18^ Arguably, this broader approach is essential as a part of any open, accountable, democratic process.^6^^,^^15^

Despite being a long-established public health measure, relatively little has been written on the ethical implications of fluoridation, from the perspective of different moral, ethical and political philosophies. The aim of this scoping review is to examine how the ethics of fluoridation has been appraised in the literature.

## Method

Given the likely breadth and nature of the available literature on the topic, a scoping review was considered appropriate to meet the objectives of the research, which were to identify: how the ethics of fluoridation has been discussed; what branches of ethics have been considered; and any knowledge gaps relating to the ethics of fluoridation. The method used a modified framework proposed by Arskey and O'Malley,^19^ with developments by the Joanna Briggs Institute,^20^^,^^21^ and Preferred Reporting Items for Systematic Reviews and Meta-analyses extension for Scoping Reviews (PRISMA ScR) (see online Supplementary Information).^22^ It comprised seven main stages: identifying the research question; searching for and identifying relevant studies; selecting relevant studies; extracting, analysing, and collating data; summarising the results; discussion; and conclusions from the findings.

### Data sources

Key terms were identified relevant to the review by undertaking an initial search. These included ‘public health ethics', ‘biomedical ethics', ‘bioethics', ‘community water fluoridation' and ‘fluoridation'. The title and abstract of articles identified were analysed for terms to develop a comprehensive search strategy and Boolean operators (AND and OR) were applied. A broad cross-disciplinary search was undertaken by adapting the search strategy (Table 1) across six electronic databases: Medline via OVID; Scopus; Web of Science; Cochrane Library; University of Sheffield StarPlus; and Google Scholar. Several other sources of information were searched, including: reference lists of articles identified from the database search; hand-searching of key journals, websites of relevant networks and organisations; and conferences. A grey literature search was undertaken on OpenGrey.

**Table 1:** Search strategy for electronic databases

**Database**	**Search terms**
Medline via Ovid Scopus Web of Science Cochrane Library Google Scholar University of Sheffield StarPlus	Ethic* OR bioethics* OR medical ethic* OR biomedical ethic* or public health ethic* AND Fluoridation OR water fluoridation OR community water fluoridation

Records obtained from database and other searches were entered into reference manager software (EndNote 20) and duplicates were removed.

### Data selection

All relevant literature considered from searches were considered with the following inclusion criteria:

- Literature including ethics and fluoridation
- Literature available electronically with full text
- Literature written in English.

**Fig. 1:**
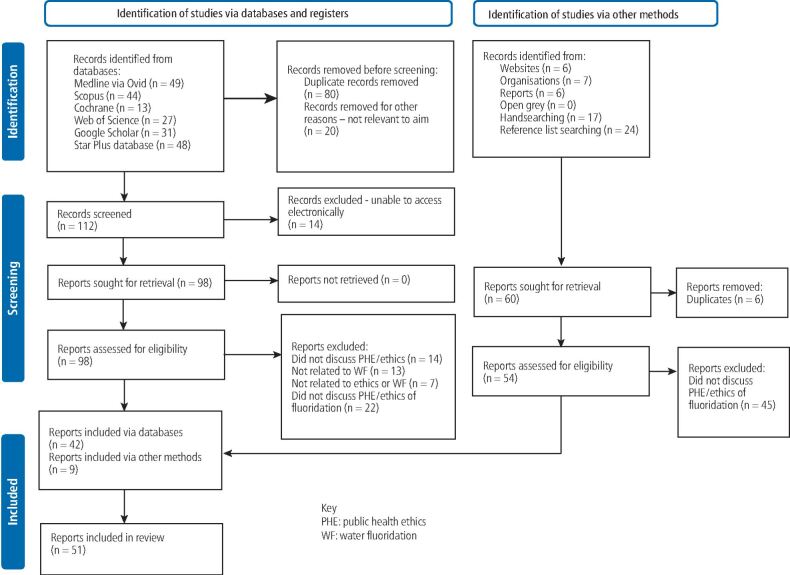
Flow diagram of the search strategy^22^

There was no restriction on literature type or study design. However, the search included literature published in the last 60 years, as it was postulated that any before this may not be relevant to contemporary populations and a preliminary search found no studies available electronically before this. Literature in languages other than English were excluded for reasons of practicality and feasibility. Literature meeting the inclusion criteria was analysed in two steps: title and abstract screening, then review of the full text, as shown in the PRISMA diagram (Fig. 1).^20^ Both authors (BP, TD) screened studies for suitability for final inclusion. Where there was doubt on the relevance to the aim of this review, this was discussed and resolved by the two authors.

### Data extraction

Relevant data were extracted and recorded in a data extraction table using Microsoft Excel by one author (BP). This included: authors; year of publication; title; journal or organisation; country of origin; source of literature; and an overview of each record, including ethical principles considered and apparent stance on fluoridation. In addition, observations of strengths and limitations of the studies were recorded.

### Data analysis and collation

Given the nature of the literature and its heterogeneity, extracted data were analysed thematically and a narrative synthesis was produced. The included studies were also summarised in table form (Table 2).

**Table 2:** Theoretical and philosophical basis of ethical appraisal of community water fluoridation

**Basis of ethical appraisal**	**Descriptor**
**Moral theories**	
Consequentialist theories	Whether an act is morally right depends only on its consequences: an act that brings about more benefit than harm is good, while one that brings more harm than benefit is not. Example: utilitarianism
Non-consequentialist theories	Morality of an act depends on more than just consequences. Actions themselves are good or bad based on whether they obey or violate moral rules, duties, or principles. Examples: deontology, rights-based theories and principlism
**Political and social philosophies**	
Liberalism	Based on the rights of the individual, liberty, consent of the governed, political equality, and equality before the law
Communitarianism	Aims to counter individualism by emphasising a balance between individual and collective goals and values, including health and wellbeing

## Results

The findings are reported in three parts. First, an overview of the included studies is provided. This is followed by a description and analysis of the moral, political and ethical principles identified in the literature. The final part categorises the included studies based on the apparent stance on fluoridation.

### Overview of included studies

Database searching (April 2024) identified 212 records. Initially, 80 duplicates were removed and a further 20 studies were removed that did not meet the inclusion criteria. Titles and abstracts of the remaining 112 records were screened, of which 70 did not meet the inclusion criteria, leaving 42 records from databases to be included. Additionally, 60 records were identified from other methods. From these, six duplicate records were removed and a further 45 were excluded, leaving nine further records to be included. In total, 51 records were included in the review (Fig. 1).

Various study designs and types of publication were included: conceptual reviews (n = 10); qualitative studies (n = 30); position statements/reviews/reports/recommendations (n = 9) and books (n = 2).

Studies from 15 countries and five continents were included: United Kingdom (UK) (n = 15); United States (USA) (n = 8); Canada (n = 8); Australia (n = 6); India (n = 2); New Zealand (n = 2); Brazil (n = 2); United Arab Emirates (UAE) (n = 1); Turkey (n = 1); South Korea (n = 1); Italy (n = 1); Chile (n = 1); Israel (n = 1); Switzerland (n = 1); and Ireland (n = 1).

### Findings by theoretical and philosophical basis

Most studies discussed the ethics of fluoridation in terms of various moral theories and political philosophies (Table 2), often citing or implying several principles, and very few discussed fluoridation in terms of one ethical principle alone. However, the stance taken was often not explicit in the papers and some adopted multiple stances. For example, Song and Kim^23^ considered principlism alongside utility and liberty and the involvement of stakeholders to encourage democratic decision-making.

#### Moral-based theories

The review identified 11 studies that were informed by or implied consequentialist theories in varying depth. For example, Atchison discussed fluoridation from a utilitarian perspective, arguing it is morally right if it prevents caries and has more value than alternative actions.^24^ The author also questioned the ethics of choosing not to act, arguing to do so would not be morally right unless caries is no longer a public health problem.

In total, 40 studies referred to or implied non-consequentialist theories. One discussed this in terms of deontological theory and held that fluoridation violates international agreements on human rights and biomedicines.^25^ Principlism and public health principlism proposes ethical principles to consider beyond merely their consequences. Beauchamp and Childress' principles for medical care^26^ of autonomy, non-maleficence, beneficence and justice were most frequently cited. A total of 36 studies discussed fluoridation in relation to these, either citing autonomy alone (n = 4),^27^^,^^28^^,^^29^^,^^30^ or arguably implying the principle of autonomy in terms such as ‘infringement of personal freedom' or ‘violation of civil or individual liberties' (n = 6).^31^^,^^32^^,^^33^^,^^34^^,^^35^^,^^36^ Furthermore, 22 discussed or implied autonomy and/or non-maleficence, beneficence and justice,^17^^,^^24^^,^^37^^,^^38^^,^^39^^,^^40^^,^^41^^,^^42^^,^^43^^,^^44^^,^^45^^,^^46^^,^^47^^,^^48^^,^^49^^,^^50^^,^^51^^,^^51^^,^^52^^,^^53^^,^^54^^,^^55^ and six referred to all four principles.^23^^,^^56^^,^^57^^,^^58^^,^^59^^,^^60^ Public health principlism was applied in six studies,^17^^,^^23^^,^^42^^,^^44^^,^^45^^,^^59^ referring to Childress *et al*.^14^ (n = 5)^17^^,^^23^^,^^42^^,^^44^^,^^45^ or Klugman's (n = 1)^61^ ethical principles.

#### Political philosophies

In total, 17 studies discussed or implied liberalism, by referring to it directly or indirectly with reference to liberty and freedom.^23^^,^^25^^,^^27^^,^^28^^,^^31^^,^^33^^,^^36^^,^^37^^,^^39^^,^^42^^,^^58^^,^^59^^,^^62^^,^^63^^,^^64^^,^^65^^,^^66^ No studies referred to communitarianism directly but five implied its principles.^36^^,^^37^^,^^51^^,^^58^^,^^60^

#### Other theories and models

Four studies considered fluoridation in terms of violation of medical ethics and the Nuremberg code.^17^^,^^33^^,^^67^^,^^68^ Ten appraised fluoridation using the Nuffield Bioethics report^17^^,^^38^^,^^42^^,^^47^^,^^52^^,^^58^^,^^63^^,^^64^^,^^66^^,^^69^ for public health measures and the stewardship model, which proposes an intervention ladder that encourages approaches that are less intrusive (lower on the ladder) and allow for public consultation in decision-making.^15^

### Findings by stance on fluoridation

Three apparent stances were identified in the literature: supporting, opposing and neutral to fluoridation (Table 3). Each is described in the next section with reference to the ethical approach cited or implied. Those studies that considered the ethics of fluoridation more extensively are summarised in more detail.

**Table 3:** Overview of included studies appraising the ethics of fluoridation

**Authors**	**Country**	**Apparent stance**	Moral and ethical principles identified*
Anand *et al.*^57^	India	Neutral	Principlism (autonomy beneficence, non-maleficence, justice)
Armfield^27^	Australia	Supportive	Freedom, liberty, principlism (autonomy)
Atchison^24^	USA	Supportive	Principlism (autonomy and beneficence), veracity and justice, utilitarianism, paternalism, distributive justice
Ateş A and Özer^51^	Turkey	Neutral	Principlism (autonomy and beneficence), ‘common good'
Awofeso^44^	Australia	Opposed	Principlism (autonomy and beneficence), public health principlism (Childress *et al.*)^14^
Awofeso *et al.*^45^	UAE	Opposed	Principlism (autonomy and beneficence), public health principlism (Childress *et al*.)^14^
Ballantyne^55^	New Zealand	Neutral	Principlism (autonomy, beneficence, justice)
Botchey *et al.*^40^	USA	Supportive	Principlism (autonomy, benefits)
Bradley *et al.*^56^	UK	Supportive	Principlism (autonomy, beneficence, non-maleficence, distributive justice)
British Fluoridation Society^58^	UK	Supportive	Principlism (autonomy, benefit, justice) avoidance of harm, common good, paternalism, liberalism, Nuffield framework^15^
CADTH^54^	Canada	Neutral	Implied principlism (autonomy, beneficence)
Calman^69^	UK	Neutral	Nuffield framework,^15^ Mill's harm principle
Cheng *et al.*^50^	UK	Neutral	Principlism (autonomy, harm, benefit)
Coggon and Cooper^65^	UK	Neutral	Liberty
Coggon and Viens^60^	UK	Neutral	Principlism (autonomy, benefits, harms, justice), implied communitarianism (common good)
Cohen and Locker^48^	Canada	Neutral	Bioethics, principlism (autonomy, beneficence)
Cross^25^	Switzerland	Opposed	Deontology, paternalism, utilitarianism, libertarianism
Cross and Carton^67^	UK	Opposed	Nuremberg code
Curtin^36^	Ireland	Supportive	Implied principlism (implied autonomy), liberty, ‘common good'
Dickinson *et al.*^43^	Canada	Supportive	Implied utilitarianism, and implied principlism (benefits, harms, individual choice)
Gannon^68^	Australia	Opposed	Nuremberg code
Garbin *et al.*^53^	Brazil	Neutral	Principlism (autonomy, non-maleficence)
Gibson *et al.*^30^	UK	Neutral	Principlism (autonomy)
Gooch^41^	USA	Supportive	Implied principlism (discuss autonomy, benefits)
Gostin and Gostin^70^	USA	Supportive	Mill's harm principle, paternalism, utility, social justice
Iheozor *et al.*^2^	UK	Neutral	Ethics of mass medication
Jiang *et al.*^63^	New Zealand	Supportive	Libertarian, utilitarian, principles approach of the Nuffield framework^15^
Kalamatianos and Narvai^49^	Brazil	Neutral	Social utility, principlism (autonomy, benefit), ‘greatest good'
Lucyk and McClaren^52^	Canada	Neutral	Principlism (autonomy, beneficence, non-maleficence), utilitarianism, Nuffield framework^15^
Klugman^61^	USA	Neutral	Public health principlism - solidarity, efficacy, integrity, and dignity
Knox et al.^34^	Australia	Neutral	Implied principlism (implied autonomy, right to choose infringement on individual rights)
Lang^66^	Canada	Neutral	Nuffield framework,^15^ liberty
Leeder^59^	Australia	Neutral	Principlism (implied autonomy, beneficence), liberty
Lennon *et al.*^38^	UK	Supportive	Nuffield framework,^15^ principlism (autonomy, benefit)
Lowery *et al.*^62^	UK	Neutral	Utilitarianism, principlism (implied autonomy (freedom of choice), benefit), ‘attack on liberty', ‘mass medicalisation'
Martin^28^	USA	Neutral	Liberty, principlism (autonomy [violation of human rights])
Mclaren and Petit^64^	Canada	Supportive	Liberty, Nuffield framework^15^
McNally and Downie^37^	Canada	Supportive	Freedom of choice, principlism (autonomy, benefit, harm), ‘common good'
Michels^74^	USA	Neutral	Utilitarianism
NICE^32^	UK	Neutral	Freedom, principlism (autonomy (violation of human rights), benefits, harms)
O'Neill *et al.*^75^	Canada	Neutral	Ethics of mass medication
Peckham and Awofeso^47^	Australia	Opposed	Principlism (benefit, harm), Nuffield framework
Pizzo *et al.*^33^	Italy	Neutral	Implied autonomy (freedom of choice), violation of medical ethics and human rights
Pratt *et al.*^31^	USA	Neutral	Implied autonomy (violation of rights), liberty
Quinteros^42^	Chile	Supportive	Liberalism versus utilitarianism, principlism (autonomy, benefit, non-maleficence), public health principlism (Childress *et al.*),^14^ principles of Nuffield framework^15^
Rajaran *et al.*^35^	India	Neutral	Implied principlism (implied autonomy - freedom of choice and infringement on individual rights)
Shakeri *et al.*^17^	UK	Neutral	Principlism (autonomy, beneficence), public health principlism (Childress *et al.*^14^), Nuffield framework,^15^ Nuremberg
Shaw^46^	UK	Opposed	Principlism (implied autonomy, [‘encroach on human rights'], harm, benefit)
Shaw^29^	UK	Neutral	Principlism (autonomy)
Song and Kim^23^	South Korea	Neutral	Principlism (autonomy, beneficence, non-maleficence, justice), utility, liberty, public health principlism (Childress *et al.*^14^)
Zusman^39^	Israel	Supportive	Freedom (liberty), principlism (benefit)
Key * = Unless otherwise indicated, ‘principlism' refers to the four biomedical principles identified by Beauchamp and Childress^26^ CADTH = Canadian Agency for Drugs and Technologies in Health NICE = National Institute for Health and Clinical Excellence			

#### Supporting fluoridation

A total of 15 studies assessed as supporting fluoridation referred to the ethical positions of principlism in terms of benefit (beneficence) and minimising harm (non-maleficence), utilitarianism (the moral duty of the policy [fluoridation] is determined by its consequences [prevention of caries] or its utility), or implied communitarianism (common good).^24^^,^^27^^,^^36^^,^^37^^,^^38^^,^^39^^,^^40^^,^^41^^,^^42^^,^^43^^,^^56^^,^^58^^,^^63^^,^^64^^,^^70^

McNally and Downie discussed autonomy, benefits and harms of fluoridation, arguing the ‘common good' should be placed above individual desires.^37^ The authors also advocated the importance of taking into consideration the voice of society, and particularly those who are disadvantaged. Similarly, Curtin argued that individual and religious liberty are not absolute, and that professional freedom is also a right, stating that fluoridation is not unduly restrictive to society and serves the temporary common good and should be lawfully regulated.^36^

Several studies used utilitarian arguments, with some referring to the principles of the Nuffield framework.^15^^,^^42^^,^^52^^,^^63^ Democratic decision-making was also seen as important in implementing public health measures and that liberal emphasis of individual autonomy should not prevent community health benefit and reduction of inequalities.^38^^,^^39^^,^^63^^,^^69^ In addition, Jiang *et al*.^63^ argued that fluoridation is not coercive as it does not require a lifestyle change when compared to alternative measures, and that the imperative of individual consent can be argued both ways: it should be required if fluoridation is to be ceased as well as introduced.

In discussing public health measures, including fluoridation, Gostin and Gostin drew on communitarian and utilitarian principles by arguing that ‘well-directed paternalism promotes a “broader freedom” for the many'.^70^ They questioned Mill's theory of liberty in public health measures, which promotes individual autonomy as essential for happiness.^71^ The authors argue that Mill promotes utility of freedom at all costs, which could be detrimental, as it may lead to more ill health, greater inequalities and a community void of shared values and spirit.^70^

In supporting fluoridation, Quinteros debated the perspective of liberalism versus utilitarianism and emphasised that bioethics has developed in countries that are economically developed with good access to information.^42^ Consequently, they argue ethical debates may not be culturally consistent or relevant to other countries where information needed for informed choice may not be accessible.

#### Opposing fluoridation

Seven studies assessed as opposing fluoridation mostly cited individual ethical principles within principlism. However, some cited moral-based consequentialism and non-consequentialist theories.^25^^,^^44^^,^^45^^,^^46^^,^^47^^,^^67^^,^^68^ For example, Cross and Carton^67^ opposed fluoridation on the basis that it violates deontological principles and individuals should not be treated as means to an end.^72^ They argued that it is part of a biased utilitarian agenda and that public opposition has led to governments using paternal libertarian approaches, such as ‘nudging' - an approach that cajoles the public to change behaviours - to enable administrations to achieve health goals.^73^

Awofeso *et al*. espoused alternative caries prevention approaches, arguing that fluoridation infringes the principle of autonomy and violates the principle of non-maleficence given dental fluorosis and other putative health risks. They concluded there is insufficient ethical justification for fluoridation in Australia.^44^^,^^45^

Two studies saw fluoride as a medicine (defining this as a substance to prevent disease) according to European and American pharmaceutical codes.^67^^,^^68^ For them, fluoridation is morally and ethically unacceptable and fails to comply with the Nuremberg code of practice and other medical codes and regulations, which propose that consumers should be fully informed of risks and benefits. In this case, they argue the public is not fully informed of what they are consuming.^67^

#### Neutral to fluoridation

In total, 29 records were assessed as neutral to fluoridation in approach, citing mixed ethical viewpoints, including principlism,^17^^,^^23^^,^^28^^,^^29^^,^^30^^,^^31^^,^^32^^,^^33^^,^^34^^,^^35^^,^^48^^,^^49^^,^^50^^,^^51^^,^^52^^,^^53^^,^^54^^,^^55^^,^^57^^,^^59^^,^^60^^,^^62^ utilitarianism,^23^^,^^49^^,^^52^^,^^62^^,^^74^ liberty,^23^^,^^28^^,^^31^^,^^59^^,^^62^^,^^65^^,^^66^ and the Nuffield approach^17^^,^^52^^,^^66^^,^^69^ in their discussions. Most were qualitative papers. The primary aim of some was to appraise ethics^17^^,^^23^^,^^29^^,^^35^^,^^48^^,^^49^^,^^50^^,^^53^^,^^55^^,^^57^^,^^59^^,^^60^^,^^61^ but others considered fluoridation more generally and in which ethics was discussed.^2^^,^^28^^,^^30^^,^^31^^,^^32^^,^^33^^,^^34^^,^^50^^,^^52^^,^^54^^,^^62^^,^^66^^,^^69^^,^^75^^,^^76^ The tension between autonomy and beneficence in public health interventions was frequently highlighted, plus the need for public debate in decision-making processes. For example, Anand *et al*.^57^ discussed all four of Beauchamp and Childress' principles^26^ and highlighted the conflict between autonomy and beneficence with fluoridation, and advocated debating ethical consideration in public health policymaking.

Cheng *et al*.^50^ argued that anti-fluoridation arguments overstated any controversies with identifying harms and seeing fluoridated water as a medicine. However, they also proposed that the UK Department of Health's funding of organisations that support fluoridation, and selective reporting of research findings, undermined its scientific independence. In addition, they highlighted the limitations of studies used to support the safety of fluoride, such as small sample sizes, and postulated that selective use of evidence can lead to public mistrust.

In a similar context, Shakeri *et al*.^17^ presented a neutral analysis of food fortification and fluoridation using two public health ethical frameworks: the justificatory approach of Childress *et al*.^14^ and the Nuffield stewardship model.^15^ They argued, although supporting fluoridation overall, that there are aspects of both frameworks that are not fulfilled. For example, if consumers are unaware of the composition of water, accusations of deception and passive consumption might be made. They also considered whether fluoride is seen as a medicine and the associated ramifications in relation to the Nuremberg code. Given its complexity, they concluded the ethics of public health interventions such as fluoridation need to be revisited regularly.^17^

Klugman ethically appraised fluoridation by applying principlism but using notions of efficacy (scientifically sound with a significant chance of meeting goals), integrity (inclusion of the community), solidarity (community comes together) and dignity (respecting the community using the least restrictive principle). Klugman proposed that efficacy may be unclear; while acknowledging its evidence of effectiveness, he questions whether it is feasible in the prevailing political and social climate. However, he saw fluoridation as favourable for solidarity (in terms of equity for all the community), and unclear for integrity (as it depends on the level of community engagement in decision-making) and dignity (as there are other, less restrictive means of fluoride delivery).^61^

Taking a different approach, Ateş and Özer^51^ considered ethical arguments related to fluoridation while reflecting on the impacts of different social, cultural and religious philosophies in Turkey. From the perspective of autonomy, they considered whether responsibility should be shared, individual, professional, or state, concluding that the crucial importance of any policy includes both ongoing engagement with the public and the transparency of government intent. In addition, they considered beneficence and maximising common good from a more utilitarian perspective but also discussed the influence of professional power in this process.^51^

## Discussion

This scoping review aimed to examine how the ethics of fluoridation has been appraised in the literature. It searched six databases, grey literature and reference lists of included studies. Overall, it identified that there is a relatively small body of literature on the topic from a range of countries that has taken different approaches to its appraisal, frequently using those intended for individual medical care rather than for public health interventions. The ethical approach taken was rarely stated and was often implied, with limited reference to moral or political theory. The literature could be categorised as appearing to be supporting, opposing, or neutral to fluoridation in its standpoint (Table 3).

Like medical ethics, public health ethics developed from bioethics.^6^ However, it differs in that it is underpinned by moral and political theories to reflect that the focus of public health is for population rather individual benefit.^6^^,^^13^ However, many included studies adopted ethical principles intended for medical care, often implicitly without reference to moral theory or ethical frameworks, which are constructed from liberal values that centre on autonomy. Arguably, these are inadequate for examining whether fluoridation is ethically justifiable, taking into account the inevitable infringement on individual consent and evidence of population benefit and risk.^57^

Consequently, a multi-dimensional approach to guide ethical decision-making in public health has been recommended, for example, using principlism or frameworks proposed for public health interventions.^10^^,^^14^^,^^15^^,^^18^^,^^60^^,^^61^^,^^76^

Utilitarianism and the ethical principle of beneficence were frequently emphasised by those supporting fluoridation. Fluoridation was discussed as a utilitarian intervention protecting population health, but the ‘liberal objection' to utilitarianism raises the argument of infringing individual liberties, yet it is seen as justified if its implementation avoids harm to others.^24^^,^^42^^,^^63^ There is evidence that fluoridation benefits oneself, as it reduces the risk of dental caries and avoids pain, discomfort and treatment, and this may include children and socioeconomically deprived and other vulnerable groups.^77^^,^^78^ However, harm to oneself and others could include the increased risk of dental fluorosis and any associated treatment costs,^79^ especially in areas where caries levels are low or topical fluoride levels are high.

Opponents of fluoridation often centre their concerns on infringement of choice and individual consent, especially if it is mandated policy.^80^ Such ethical challenges can become highly emotive in the literature and vociferously argued as against human rights by anti-fluoridation activists.^46^^,^^67^^,^^68^^,^^81^ The Nuremberg code is also commonly cited in anti-fluoridation literature as fluoride is viewed as a medicine and hence, in this conception, fluoridation is seen as mass medication.^67^^,^^68^ The controversy arises as the consumer may not be fully informed of this addition and therefore is unable to consent. Others argue that fluoride is not a medicine as it occurs naturally in similar concentrations to those in fluoridation schemes.^50^ Although this debate is beyond the scope of this review, legislation and medicines regulatory bodies ultimately define medicines and their use, and this may vary internationally and be subject to change. In the UK, the Medicines and Healthcare products Regulatory Agency licences medicinal products and has stated that fluoride in water is outside its remit as fluoride forms part of the diet and does not regard it a medicinal product.

Authors adopting a more neutral standpoint on fluoridation often used a combination of principles and frameworks, including utilitarianism, principlism and public health principalism.^10^^,^^17^^,^^23^^,^^26^^,^^49^^,^^52^^,^^57^^,^^59^^,^^61^^,^^62^ Discussions centred on the conflicts of the different ethical principles to each other on fluoridation, for example, autonomy (fluoride as mass medication) opposing the communal positive effects (beneficence).^57^^,^^62^ The use of frameworks specifically intended for public health has been recommended to address these challenges and to advance ethical appraisal,^60^^,^^76^ particularly by shifting the focus away from individual focus toward public benefit, trust, proportionality and accountability, and judicious use of research data.^55^ However, concerns have been expressed about the power of science in such assessments. Martin argues that fluoridation supporters can dominate debates, particularly when supported by evidence of effectiveness, funding and professional accreditations, and risk overwhelming other potentially legitimate arguments.^28^^,^^82^ A related concern is risk of unconscious or conscious bias in studies evaluating fluoridation that are led by dental public health specialists, the findings of which influence ethical decision-making.^27^^,^^47^^,^^83^ This reinforces the view of the Nuffield Council on Bioethics who asserted: ‘the only ethical solution in a liberal democracy is to continue a public debate which aims to be transparent and should be undertaken with scientific objectivity, rigour and integrity so a balanced decision can be reached'.^15^

Although this review followed methodological frameworks and comprehensively searched the literature, some relevant studies may have been missed, including any published in languages other than English. In addition, literature was included on the basis of relevance rather than quality, with the latter not being formally assessed. Consequently, the review may provide breadth but not depth of analysis. A related problem was that studies frequently implied moral, political and ethical theory, and principles of these were interpreted subjectively, including their apparent stance on fluoridation, and therefore may misrepresent the intended meaning.

## Conclusion

A range of approaches have been taken to appraise the ethics of fluoridation in the literature and these were frequently conceived for individual medical rather than public health interventions. Consequently, they often emphasise certain principles or theories that may suit a particular stance but are inadequate to resolve tension between inevitable infringement of individual consent and collective benefit in public health. Other approaches conceived specifically for public health interventions exist and they have more utility in debates and ethical decision-making.

## Supplementary Information

PRISMA Checklist (PDF 187KB)

## Data Availability

Any data included in the study are available in the cited papers.

## References

[1] NHS Centre for Reviews and Dissemination. A systematic review of public water fluoridation. 200. Available at https://www.york.ac.uk/media/crd/crdreport18.pdf (accessed August 2023).

[2] Iheozor-Ejiofor Z, Worthington H V, Walsh T *et al*. Water fluoridation for the prevention of dental caries. *Cochrane Database Syst Rev* 2015; DOI: 10.1002/14651858.CD010856.pub2. 10.1002/14651858.CD010856.pub2 PMC6953324 26092033

[3] UK Government. A rapid review of evidence on the cost-effectiveness of interventions to improve the oral health of children aged 0-5 years. 2016. Available at https://assets.publishing.service.gov.uk/media/5a802b7be5274a2e8ab4e970/Rapid_review_ROI_oral_health_5_year_old.pdf (accessed June 2024).

[4] Duane B, Lyne A, Parle R, Ashley P. The environmental impact of community caries prevention - part 3: water fluoridation. *Br Dent J* 2022; **233:** 303-307. 10.1038/s41415-022-4251-5 36028695

[5] UK Government. Water fluoridation: health monitoring report for England 2022. 2022. Available at https://www.gov.uk/government/publications/water-fluoridation-health-monitoring-report-for-england-2022 (accessed September 2023).

[6] Holland S. *Public health ethics*. 2nd ed. Cambridge: Polity Press, 2015.

[7] Acheson D. Public health in England: the report of the Committee of Inquiry into the Future Development of the Public Health Function. 1988. Available at https://archive.org/details/b32220509/page/0/mode/2up (accessed August 2023).

[8] Miles A, Loughlin M. Philosophy, freedom and the public good: a review and analysis of ‘Public Health Ethics', Holland S (2007). *J Eval Clin Pract* 2009; **15:** 838-858.

[9] Dawson A, Verweij M F. *Ethics, prevention, and public health: issues in biomedical ethics*. Oxford: Clarendon Press, 2007.

[10] Kass N E. An ethics framework for public health. *Am J Public Health* 2001; **91:** 1776-1782. 10.2105/ajph.91.11.1776 PMC1446875 11684600

[11] Have M T, van der Heide A, Mackenbach J P, De Beaufort I D. An ethical framework for the prevention of overweight and obesity: a tool for thinking through a programme's ethical aspects. *Eur J Public Health* 2013; **23:** 299-305. 10.1093/eurpub/cks052 23132871

[12] Dawson A. Theory and practice in public health ethics: a complex relationship. *In* Peckham S, Hann A, (eds) *Public health ethics and practice*. pp 191-209. Bristol: Policy Press, 2010.

[13] Patel B G, Patrick A, Dyer T A. The ethics of community water fluoridation: Part 1 - an overview of public health ethics. *Br Dent J* 2025; **238:** 311-315. 10.1038/s41415-024-8058-4 PMC11908967 40087432

[14] Childress J F, Faden R R, Gaare R D *et al.* Public health ethics: mapping the terrain. *J Law Med Ethics* 2002; **30:** 170-178. 10.1111/j.1748-720x.2002.tb00384.x 12066595

[15] Nuffield Council On Bioethics. Public health: ethical issues. 2007. Available at https://www.nuffieldbioethics.org/assets/pdfs/Public-health-ethical-issues.pdf (accessed July 2023).

[16] National Collaborating Centre for Healthy Public Policy. ‘Principlism' and frameworks in public health ethics. 2016. Available at https://ccnpps-ncchpp.ca/principlism-and-frameworks-in-public-health-ethics/ (accessed August 2023).

[17] Shakeri A, Adanty C, Kugathsan H. Revisiting the ethical framework governing water fluoridation and food fortification. *Clin Ethics* 2020; **15:** 175-180.

[18] Tannahill A. Beyond evidence - to ethics: a decision-making framework for health promotion, public health and health improvement. *Health Promot Int* 2008; **23:** 380-390. 10.1093/heapro/dan032 18971394

[19] Arksey H, O'Malley L. Scoping studies: towards a methodological framework. *Int J Soc Res Method* 2005; **8:** 19-32.

[20] Peters M D J, Godfrey C M, Khalil H, McInerney P, Parker D, Soares C B. Guidance for conducting scoping reviews. *Int J Evid Based Healthc* 2015; **13:** 141-146. 10.1097/XEB.0000000000000050 26134548

[21] Khalil H, Bennett M, Godfrey C, McInerney P, Munn Z, Peters M. Evaluation of the JBI scoping reviews methodology by current users. *Int J Evid Based Healthc* 2020; **18:** 95-100. 10.1097/XEB.0000000000000202 31567603

[22] Page M J, McKenzie J E, Bossuyt P M *et al.* The PRISMA 2020 statement: an updated guideline for reporting systematic reviews. *Syst Rev* 2021; **10:** 89. 10.1186/s13643-021-01626-4 PMC8008539 33781348

[23] Song Y, Kim J. Community water fluoridation: caveats to implement justice in public oral health. *Int J Environ Res Public Health* 2021; **18:** 2372. 10.3390/ijerph18052372 PMC7967766 33804357

[24] Atchison K A. The ethical issues of fluoridation. *J Am Coll Dent* 1992; **59:** 14-17. 1401578

[25] Cross D. An unhealthy obsession with fluoride. *Nanotechnol Percept* 2015; **11:** 169-185.

[26] Beauchamp T L, Childress J F. *Principles of biomedical ethics*. 7th ed. New York: Oxford University Press, 2013.

[27] Armfield J M. When public action undermines public health: a critical examination of antifluoridationist literature. *Aust N Z Health Policy* 2007; **4:** 25. 10.1186/1743-8462-4-25 PMC2222595 18067684

[28] Martin B. *Scientific knowledge in controversy: the social dynamics of the fluoridation debate*. New York: SUNY Press, 1991.

[29] Shaw D. Neuroenhancing public health. *J Med Ethics* 2014; **40:** 389-391. 10.1136/medethics-2012-101300 23793059

[30] Gibson L B, Blake M, Baker S. Inequalities in oral health: the role of sociology. *Community Dent Health* 2016; **33:** 156-160. 27352473

[31] Pratt E Jr, Rawson R D, Rubin M. Fluoridation at fifty: what have we learned? *J Law Med Ethics* 2002; **30:** 117-121. 12508513

[32] NICE Citizens Council. *Mandatory public health measures*. London: National Institute for Health and Care Excellence, 2005. 28230959

[33] Pizzo G, Piscopo M R, Pizzo I, Giuliana G. Community water fluoridation and caries prevention: a critical review. *Clin Oral Investig* 2007; **11:** 189-193. 10.1007/s00784-007-0111-6 17333303

[34] Knox M C, Garner A, Dyason A, Pearson T, Pit S W. Qualitative investigation of the reasons behind opposition to water fluoridation in regional NSW, Australia. *Public Health Res Pract* 2017; **27:** 2711705. 10.17061/phrp2711705 28243671

[35] Rajarajan G, Kumar R P, Priyadorshini S P. A review on the ethics of artificial water fluoridation. *Drug Invent Today* 2019; **11:** 102-107.

[36] Curtin J. The ethical aspect of fluoridation. *J N J State Dent Soc* 1966; **37:** 374-377. 5220306

[37] McNally M, Downie J. The ethics of water fluoridation. *J Can Dent Assoc* 2000; **66:** 592-593. 11253350

[38] Lennon M A, Beal J F, Rugg-Gunn A J. Do we let children's teeth decay just because some people object to topping up the natural fluoride that's already in our water? *Community Dent Health* 2008; **25:** 66-69. 18637316

[39] Zusman S P. Water fluoridation in Israel: ethical and legal aspects. *J Public Health Rev* 2012; **34:** 1-14.

[40] Botchey S A, Ouyang J, Vivekanantham S. Global water fluoridation: what is holding us back? *Altern Ther Health Med* 2015; **21:** 46-52. 26026144

[41] US Department of Health and Human Services Federal Panel on Community Water Fluoridation. US public health service recommendation for fluoride concentration in drinking water for the prevention of dental caries *Public Health Rep* 2015; **130:** 318-331. 10.1177/003335491513000408 PMC4547570 26346489

[42] Quinteros M E. Bioethical considerations about water fluoridation: a critical review. *J Oral Res* 2016; **5:** 200-206.

[43] Dickinson J A, Guichon J, Wadey W, Da Silva K. Family physicians as advocates for community water fluoridation. *Can Fam Physician* 2023; **69:** 314-318. 10.46747/cfp.6905314 PMC10177653 37173001

[44] Awofeso N. Ethics of artificial water fluoridation in Australia. *Public Health Ethics* 2012; **5:** 161-172.

[45] Awofeso N, El Sergani M, Moussa M. Artificial water fluoridation: ethical and disease prevention implications. *Transformation Better Healthc Environ* 2014: **5:** 22.

[46] Shaw D. Weeping and wailing and gnashing of teeth: the legal fiction of water fluoridation. *Med Law Int* 2012; **12:** 11-27.

[47] Peckham S, Awofeso N. Water fluoridation: a critical review of the physiological effects of ingested fluoride as a public health intervention. *Sci World J* 2014; DOI: 10.1155/2014/293019. 10.1155/2014/293019 PMC3956646 24719570

[48] Cohen H, Locker D. The science and ethics of water fluoridation. *J Can Dent Assoc* 2001; **67:** 578-580. 11737979

[49] Kalamatianos P A, Narvai P C. Ethical aspects of the use of fluoride products in Brazil: a view of public health policy formulators. *Ciênc Saúde Colet* 2006; **11:** 63-69.

[50] Cheng K K, Chalmers I, Sheldon T A. Adding fluoride to water supplies. *BMJ* 2007; **335:** 699-702. 10.1136/bmj.39318.562951.BE PMC2001050 17916854

[51] Ateş A, Özer Ç. Ethical approach to fluoridation in drinking water systems of UK and Turkey. *J Agric Environ Ethics* 2017; **30:** 171-178.

[52] Lucyk K, McLaren L. Is the future of ‘population/public health' in Canada united or divided? Reflections from within the field. *Health Promot Chronic Dis Prev Can* 2017; **37:** 223-227. 10.24095/hpcdp.37.7.03 PMC5650033 28703704

[53] Garbin C A S, Santos L F Pd Garbin A J I, Moimaz S A S, Saliba O. Fluoridation of public water supply: bioethical, legal and political approach. *Rev Bioét* 2017; **25:** 328-337.

[54] Canadian Agency for Drugs and Technologies in Health Technology Review. Community water fluoridation programs: a health technology assessment - environmental assessment. 2019. Available at https://caphd.ca/wp-content/uploads/2024/11/ht0022-cwf-environmental-report.pdf (accessed April 2024).

[55] Ballantyne A. Adjusting the focus: a public health ethics approach to data research. *Bioethics* 2019; **33:** 357-366. 10.1111/bioe.12551 30667080

[56] Bradley P M, Burls A. *Ethics in public and community health*. 1st ed. Hoboken: Taylor and Francis, 2012.

[57] Anand K, Baridalyne N, Moorthy D, Kapoor S K, Sankar R, Pandav C S. Ethical issues in public health policy. *Nat Med J India* 2002; **15:** 97-100. 12044125

[58] The British Fluoridation Society. Extent of water fluoridation. 2020. Available at https://bfsweb.org/extent/ (accessed September 2023).

[59] Leeder S R. Ethics and public health. *Int Med J* 2004; **34:** 435-439. 10.1111/j.1445-5994.2004.00643.x 15271180

[60] UK Government. Public health ethics in practice. 2017 Available at https://www.gov.uk/government/publications/public-health-ethics-in-practice (accessed April 2024).

[61] Klugman C M. Public health principlism. *J Health Ethics* 2007; **4:** 1-29.

[62] Lowery G, Flinders M, Gibson B J. When evidence alone is not enough: the problem, policy and politics of water fluoridation in England. *Evid Policy* 2021; **17:** 507-523.

[63] Jiang Y, Foster Page L A, McMillan J, Lyons K, Broadbent J, Morgaine K C. Is New Zealand water fluoridation justified? *N Z Med J* 2014; **127:** 80-86. 25447252

[64] McLaren L, Petit R. Universal and targeted policy to achieve health equity: a critical analysis of the example of community water fluoridation cessation in Calgary, Canada in 2011. *Crit Public Health* 2018; **28:** 153-164.

[65] Coggon D, Cooper C. Fluoridation of water supplies. Debate on the ethics must be informed by sound science. *BMJ* 1999; **319:** 269-270. 10.1136/bmj.319.7205.269 PMC1126914 10426716

[66] Lang R. *Exploring parental views on community water fluoridation and alternative policy options in the context of cessation*. Calgary: University of Calgary, 2018. Master's thesis.

[67] Cross D W, Carton R J. Fluoridation: a violation of medical ethics and human rights. *Int J Occup Environ Health* 2003; **9:** 24-29. 10.1179/107735203800328830 12749628

[68] Gannon M. Hypocrisy on tap. 2017. Available at https://fluoridationqueensland.com/fluoridation-hypocrisy-on-tap-michael-gannon/ (accessed August 2023).

[69] Calman K. Beyond the ‘nanny state': stewardship and public health. *Public Health* 2009; DOI: 10.1016/j.puhe.2008.10.025. 10.1016/j.puhe.2008.10.025 PMC7118790 19135693

[70] Gostin L O, Gostin K G. A broader liberty: JS Mill, paternalism and the public's health. *Public Health* 2009; **123:** 214-221. 10.1016/j.puhe.2008.12.024 19249800

[71] Mill J S. *On liberty, utilitarianism, and other essays*. Oxford: Oxford University Press, 2015.

[72] Coughlin S S. Ethical issues in epidemiologic research and public health practice. *Emerg Themes Epidemiol* 2006; **3:** 16. 10.1186/1742-7622-3-16 PMC1594564 17018147

[73] Thaler R H, Sunstein C R. *Nudge: improving decisions about health, wealth and happiness*. London: Penguin, 2009.

[74] Michels K B. A maternalistic approach to prevention. *Int J Epidemiol* 2005; **34:** 3-4. 10.1093/ije/dyh391 15649958

[75] O'Neill B, Kapoor T, McLaren L. Politics, science, and termination: a case study of water fluoridation policy in Calgary in 2011. *Rev Policy Res* 2018; **36:** 99-120.

[76] Upshur R E. Principles for the justification of public health intervention. *Can J Public Health* 2002; **93:** 101-103. 10.1007/BF03404547 PMC6979585 11968179

[77] Burt B A. Fluoridation and social equity. *J Public Health Dent* 2002; **62:** 195-200. 10.1111/j.1752-7325.2002.tb03445.x 12474623

[78] Broomhead T, Rodd H D, Baker S R *et al*. A rapid review of variation in the use of dental general anaesthetics in children. *Br Dent J* 2020; **229:** 31-39. 10.1038/s41415-020-1846-6 32651519

[79] Edmunds W M, Smedley P L. Fluoride in natural waters. *In* Selinus O (ed) *Essentials of medical geology.* pp 311-336. Netherlands: Springer, 2012.

[80] Armfield J M, Akers H F. Risk perception and water fluoridation support and opposition in Australia. *J Public Health Dent* 2010; **70:** 58-66. 10.1111/j.1752-7325.2009.00144.x 19694932

[81] Warren J A, Pain G. A complete waste of money! Water fluoridation costs for England 2013-2021. 2017. Available at https://www.researchgate.net/publication/319460176_A_Complete_Waste_of_Money_Water _Fluoridation_Costs_for_England_2013-2021 (accessed September 2023).

[82] Martin B. The sociology of the fluoridation controversy. *Sociol Quart* 1989; **30:** 59-76.

[83] Wilson P M, Sheldon T A. Muddy waters: evidence-based policy making, uncertainty and the ‘York review' on water fluoridation. *Evid Policy* 2006; **2:** 321-331.

